# Surgical outcomes of re-excimer laser phototherapeutic keratectomy (re-PTK)

**DOI:** 10.1038/s41598-021-91121-6

**Published:** 2021-06-01

**Authors:** Osamu Hieda, Chie Sotozono, Yo Nakamura, Koichi Wakimasu, Shigeru Kinoshita

**Affiliations:** 1 Department of Ophthalmology, Kyoto Prefectural University of Medicine, 465 Kajii-cho, Hirokoji-agaru, Kawaramachi-dori, Kamigyo-ku, Kyoto, 602-0841 Japan; 2 Baptist Eye Institute, Kyoto, Japan; 3 Department of Frontier Medical Science and Technology for Ophthalmology, Kyoto Prefectural University of Medicine, Kyoto, Japan

**Keywords:** Corneal diseases, Outcomes research

## Abstract

To assess the surgical outcomes of re-excimer laser phototherapeutic keratectomy (re-PTK) for recurrent disease after initial PTK. Retrospective cohort study with historical comparison group. This study involved 56 patients who underwent re-PTK (mean follow-up period: 47.9 ± 36.2 months) at the Baptist Eye Institute, Kyoto, Japan. In all subjects, corrected-distance visual acuity (CDVA) before and after re-PTK was compared. Postoperative recurrence of corneal lesion with a decrease of CDVA of two lines or more was compared with postoperative best CDVA deemed as a significant relapse. The Kaplan–Meier method was used to compare the recurrence rate post-re-PTK with that after the initial PTK. The disease classification in the 78 eyes was heterozygous-type GCD (hetero-GCD, n = 47 eyes), homozygous-type GCD (homo-GCD, n = 13 eyes), band keratopathy (n = 7 eyes), lattice corneal dystrophy (n = 6 eyes), and other (n = 5 eyes). After re-PTK, homo-GCD recurred statistically significantly earlier than hetero-GCD (*P* = 0.0042). No significant difference was found in the recurrence rate for all diseases between post-re-PTK and post initial PTK (*P* > 0.05). Surgical outcomes after re-PTK were nearly equal to those after initial-PTK.

## Introduction

Phototherapeutic keratectomy (PTK) is a surgical treatment for anterior corneal disease that involves the use of a 193-nm-wavelength argon fluoride excimer laser^1^. PTK can ablate superficial corneal opacification, such as in corneal dystrophy (CD) or corneal degeneration^2,3^. It has been reported that in some pathologies, such as Reis-Bücklers CD^4^ and homozygous granular CD (GCD) type II (GCD2)^5^, early recurrence of the disease can occur following PTK. Although cases of heterozygous GCD are often treated with PTK, they can gradually recur with a relatively slow decrease of visual acuity (VA)^4,6^. However, the recurrence of band keratopathy (BK) and gelatinous drop-like CD after PTK is rare^6^.

To date, there have been a few reported case studies on the outcomes of PTK after the recurrence of the corneal pathology post initial-PTK^7,8^ and keratoplasty^9,10^. A few previous studies have reported adverse events resulting from PTK being performed multiple times^11,12^, however, and to the best of our knowledge, there have been no published reports on the differences in the recurrence rate between the initial PTK and a re-excimer laser PTK (re-PTK).

Since 1998, our group has been performing re-PTKs, and we feel that no change between the improvement in VA and recurrence rate after re-PTK compared with the initial PTK. Thus, the purpose of this retrospective study was to investigate and assess the surgical outcomes and statistically analyze the rate of disease recurrence following re-PTK.

## Methods

This retrospective cohort study involved patients who underwent re-PTKs at the Baptist Eye Institute, Kyoto, Japan between October 1998 and May 2015 for the treatment of disease recurrence following the initial PTK. Exclusion criteria included cases of re-PTKs with a follow-up period of less than 1 month and re-PTKs with the first PTK institute unknown. The study protocols were approved by the Institutional Review Board of Kyoto Prefectural University of Medicine and the Institutional Review Board of the Baptist Eye Institute, and in accordance with the tenets set forth in the Declaration of Helsinki. The possibility of using the data was explained to the patient at the time of the surgery and the patient's consent was obtained.

The indications for re-PTK included a decrease in corrected-distance VA (CDVA) or reported episodes of pain, and the recurrence of the original disease following the initial PTK. Disease diagnoses were performed via clinical observation using slit-lamp microscopy.

In this study, the historical comparison group consisted of patients who underwent initial PTK for heterozygous-type GCD (hetero-GCD), BK, LCD, recurrent erosion, bullous keratopathy, amyloid deposition, and Reis-Bücklers CD at the Baptist Eye Institute between August 1998 and March 2010. We previously reported the postoperative outcomes of initial PTK, including the cases in this study^6^. The initial PTKs were performed for the cases of homo-GCD seen at Kyoto Prefectural University of Medicine and the Baptist Eye Institute between October 1993 and October 2006.

In all patients, CDVA was measured via Landolt C charts at a distance of 5 m, and the logarithm of the minimum angle of resolution (logMAR) CDVA was assessed. Corneal clouding and disease recurrence was diagnosed based on the slit-lamp microscopy findings. Moreover, CDVA measurement and slit-lamp examination of the patients was performed prior to re-PTK and at 1-, 3-, and 6-months postoperatively, with continued follow-up examines then being performed semi-annually. The postoperative CDVA was the highest value of CDVA recorded after re-PTK. When cataract surgery was performed within 6-months post-re-PTK, the highest CDVA after cataract surgery was regarded as the postoperative CDVA.

At all follow-up examinations, any adverse events were noted. The postoperative hyperopic shift was calculated among the eyes with a preoperative CDVA of 0.52 logMAR or more. We took difference between preoperative refractive spherical equivalent, and 3-months-postoperative refractive error without cataract surgery cases after re-PTK.

### Surgical technique

PTK was performed using one of the following three commercially-available excimer laser systems: (1) EC-5000 (Nidek Co. Ltd., Gamagori, Japan), (2) VISX Star S4 IR**®** (Johnson & Johnson Vision Care Inc., Jacksonville, Florida, USA), and (3) Technolas**®** 217z (Bausch & Lomb, Rochester, New York, USA). The details of the laser settings have been reported previously^6^. Briefly, at 3-days prior to the PTK procedure being performed, all patients received 0.5% cefmenoxime hydrochloride eye drops (Bestron®; Senju Pharmaceutical Co. Ltd., Osaka, Japan) 4-times daily and 100 mg cefcapene pivoxil hydrochloride hydrate (Flomox®; Shionogi & Co., Ltd., Osaka, Japan) orally 3-times daily. On the day of PTK, the epithelium was directly removed by excimer laser, and the ablation continued into the corneal stroma until the pathological cornea was removed under topical anesthesia. In cases in which an irregularity was prominent on the resected surface, the corneal surface was wetted with eye drops and laser ablation was performed to smooth it out. After laser ablation, the corneal bed was left at a thickness of at least 250 μm. Post PTK, all patients were initially prescribed 0.1% fluorometholone eye drops (Flumetholon®; Santen Pharmaceutical Co., Ltd., Osaka, Japan) and antibiotic eye drops 4-times daily, with the dosage tapered off over the following 12 weeks. Each patient wore a continuous-use soft contact lens on the operated cornea until the epithelial defect closed.

### Statistical analysis

The average CDVA before and after re-PTK was compared by use of the paired *t*-test. In accordance with the previous study^4,6^, the definition of the recurrence of the corneal pathology is different in relation to the type of corneal disease. As for the corneal diseases without recurrent erosion, the recurrence was defined by the level of increased opacification at the superficial layer of the central cornea that was associated with the decrease in the CDVA of 0.2 or more logMAR. When cataract progression occurred during the post-re-PTK period, cataract surgery was performed, and if the CDVA decrease remained at 0.2 or more logMAR lower than the highest CDVA after re-PTK, we identified it as a recurrence. As for the recurrent erosion, the recurrence was defined by the reported episodes of pain.

Recurrence rates after re-PTK were compared between the 5 groups, and a Cox proportional-hazards model was constructed using the explanatory variables, such as the disease groups, age, sex, laterality, ablation depth, excimer laser models, and preoperative CDVA. Some of the hazard ratios were corrected based on the Firth's penalized partial likelihood criteria^13^. Recurrence rates after re-PTK were compared with the recurrence rates after the initial PTK on a disease-by-disease basis using the Kaplan–Meier method and the log-rank test. JMP version 13.2.1 Statistical Software for Windows (SAS Institute Inc., Cary, North Carolina, USA) was used for statistical analysis, and a *P*-value of < 0.05 was considered statistically significant.

### Ethical approval

This retrospective review of patient data was approved by the Human Studies Committee of Kyoto Prefectural University of Medicine.

## Results

This retrospective study involved a total of 78 eyes of 55 subjects (34 eyes of 24 males; 44 eyes of 31 females). The mean (± standard deviation) patient age was 64.4 ± 17.9 years (range, 10–89 years), and the mean follow-up period was 47.9 ± 36.2 months (range, 1–144 months). The average ablation depth setting was 114.7 ± 31.3μm (range, 30–250 μm), and the ablation settings contained smoothing ablation; the real ablation depth was smaller than 200μm. The average elapsed time from first PTK to re-PTK was 97.2 ± 53.3 months (range, 7–244 months), and 17 eyes (21.8%) underwent cataract surgery after re-PTK.

Of the patients in Japan with GCD, 80% to 90% are reportedly afflicted with GCD2^14,15^. The appearance of heterozygous-type GCD2 is superficial whitish round patches and translucent short dash-like linear or dot-like deposits in the stroma. Dashes in GCD2 appear whiter and rarely cross each other, and these signs can be distinguished from the lattice lines in lattice CD (LCD). GCD type 1 has characteristic granules and is easily distinguishable from LCD. Homozygous-type GCD (homo-GCD) is characterized by strong opacity and rapid progression from early childhood^15^. The homo-GCD patients in this study were diagnosed based on characteristic clinical appearance, family history, age at disease onset, and/or genetic analysis.

The patients were divided into the following 5 groups: (1) hetero-GCD (32 subjects, 47 eyes), (2) homo-GCD (10 subjects, 13 eyes), (3) BK (5 subjects, 7 eyes), (4) LCD (4 subjects, 6 eyes), and (5) other (5 subjects, 5 eyes). The 'other' cases contained two subjects with amyloid deposits, recurrent erosion, bullous keratopathy and Reis-Bücklers CD. The characteristics of the patients and the depth of ablation, the follow-up period, the number of cataract surgeries performed after re-PTK, and the change in VA before and after re-PTK are listed separately by each group in Table 1. Among those cases, one-third (10/32) of the hetero-GCD cases and one-half (5/10) of the homo-GCD cases were examined via genetic analysis. All analyses were compatible to the clinical diagnoses, and all genetically diagnosed GCD cases were GCD2.

**Table 1:** Re-Excimer laser phototherapeutic keratectomy: preoperative diagnoses, corrected distance visual acuity and related characteristics.

	Mean age (years)	Gender (F/M) (eyes)	The time between re-PTK and first-PTK (months)	Ablation depth (µm)	Mean follow-up (months)	Cat-op (cases)	CDVA (LogMAR)		2 lines or more improved (%)	2 lines or more worse (%)
							Pre	Post		
Hetero-type granular corneal dystrophy	67 ± 13.1 (25–89)	26/21	119.2 ± 45.5 (49–244)	111.5 ± 15.5 (66–151)	46.6 ± 34.8 (1–120)	15	0.48 ± 0.41	0.21 ± 0.23	51	2.1
Homo-type granular corneal dystrophy	46.6 ± 24.4 (10–81)	8/5	82.8 ± 44.4 (31–206)	152.2 ± 43.1 (101.4–250)	58.6 ± 33.8 (18–126)	0	1.15 ± 0.46	0.31 ± 0.31	100	0
Band keratopathy	74 ± 9.4 (60–84)	5/2	36.3 ± 29.1 (13–95)	96.7 ± 34.9 (50–150)	27.9 ± 29.1 (3–78)	1	0.61 ± 0.64	0.37 ± 0.62	42.9	0
Lattice corneal dystrophy	67 ± 12.1 (49–78)	1/5	77 ± 31.0 (33–107)	96.5 ± 7.2 (82–100)	38.4 ± 15.7 (60–144)	0	0.90 ± 0.37	0.30 ± 0.27	83.3	0
Others	51.6 ± 22.3 (19–74)	4/1	22.8 ± 21.6 (7–60)	110.4 ± 66.6 (30–200)	14.6 ± 13.6 (1–36)	1	0.46 ± 0.78	0.38 ± 0.56	80	0
Total	64.4 ± 17.9 (10–89)	44/34	96.3 ± 52.5 (7–244)	115.7 ± 32.6 (30–250)	47.9 ± 36.2 (1–144)	17	0.66 ± 0.52	0.26 ± 0.32	64.1	1.3

The postoperative CDVA = the best CDVA recorded after PTK. Cat-op = the eyes in which cataract surgery was performed post PTK.

Ablation Depth = the mean calculated total ablation depth (epithelium and stroma). () = range.

The profiles of the initial PTK group are shown in Table 2; i.e., hetero-GCD (229 subjects, 376 eyes), homo-GCD (8 subjects, 11 eyes), BK (167 subjects, 238 eyes), LCD (8 subjects, 13 eyes), and other (20 subjects, 22 eyes). In this study, the homo-GCD included 5 eyes post penetrating keratoplasty (PK) and the homo-GCD in initial PTK group also included 5 post PK eyes, and no diseases other than the homo-GCD included eyes post PK.

**Table 2:** Initial-excimer laser phototherapeutic keratectomy group: preoperative background.

	Mean age (years)	Gender (F/M) (eye)
Hetero-type granular corneal dystrophy	66.2 ± 11.8 (21–89)	253/123
Homo-type granular corneal dystrophy	37.1 ± 24.3 (5–76)	6/5
Band keratopathy	72.9 ± 12.2 (6–101)	180/58
Lattice corneal dystrophy	51.1 ± 15.6 (33–76)	6/7
Others; 6 amyloid deposite, 9 recurrent erosion, 6 bullous keratopathy and one Reis-Buckler corneal dystrophy	51.1 ± 20.1 (9–81)	17/5

**Figure 1:**
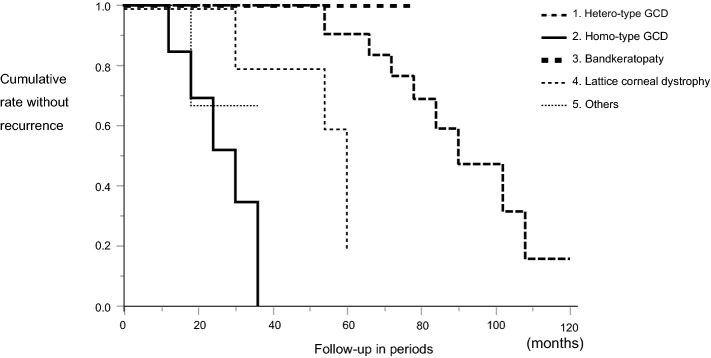
Graph showing the survival curve of the non-recurrence of disease after re-excimer laser phototherapeutic keratectomy among 5 groups constructed via the Kaplan–Meier method.

The rate of disease recurrence after re-PTK is shown in Fig. 1. The difference in recurrence rate among the 5 groups was statistically significant (log-rant test, *P* = 0.0004). The univariate Cox proportional-hazard model was constructed using age, gender, laterality, preoperative CDVA, ablation depth, laser type, and disease type. The gender and laterality were not significantly influenced by the recurrence rate. The multivariate Cox proportional-hazard model was constructed using age, preoperative CDVA, ablation depth, laser type, and disease type. Only disease type (homo/hetero) was found to be a significant variable influencing the recurrence rate (Table 3). Our findings revealed that post-re-PTK, homo-GCD recurred at a faster rate than hetero-GCD.

**Table 3:** The cox proportional hazard model of univariable and multivariable for disease recurrence.

	Univariable			Multivariable		
	HR	95% CI	P value	HR	95% CI	P value
Age	0.975	0.956–0.997	0.03*	0.992	0.962–1.023	0.60
Sex (female/male)	0.733	0.315–1.716	0.47			
Eye (OS/OD)	1.262	0.561–2.812	0.57			
Pre-CDVA	0.141	0.062–0.316	< 0.0001*	0.374	0.111–1.263	0.11
Ablation depth	1.022	1.009–1.034	0.001*	1.004	0.986–1.022	0.68
**Laser type**			0.009*			0.60
VISX/EC-5000	0.351	0.099–0.983	0.046*	0.487	0.181–2.645	0.33
T-217/EC-5000	0.225	0.052–0.671	0.006*	0.834	0.090–1.842	0.78
**Disease type**			0.0004*			0.06
Homo/hetero	92.167	12.409–684.588	< 0.0001*	27.877	2.852–272.465	0.0042*
Band/hetero	0.986	0.047–20.569	0.99	0.76	0.03–19.35	0.87
Lattice/hetero	9.991	1.993–50.087	0.0051*	4.69	0.821–26.806	0.08
Others/hetero	49.517	3.824–641.146	0.0028*	13.27	0.66–266.681	0.09

Multivariable model is adjusted for age, pre-CDVA, ablation depth, laser type and disease type.

*CI* confidence interval.

*P < 0.05.

**Figure 2:**
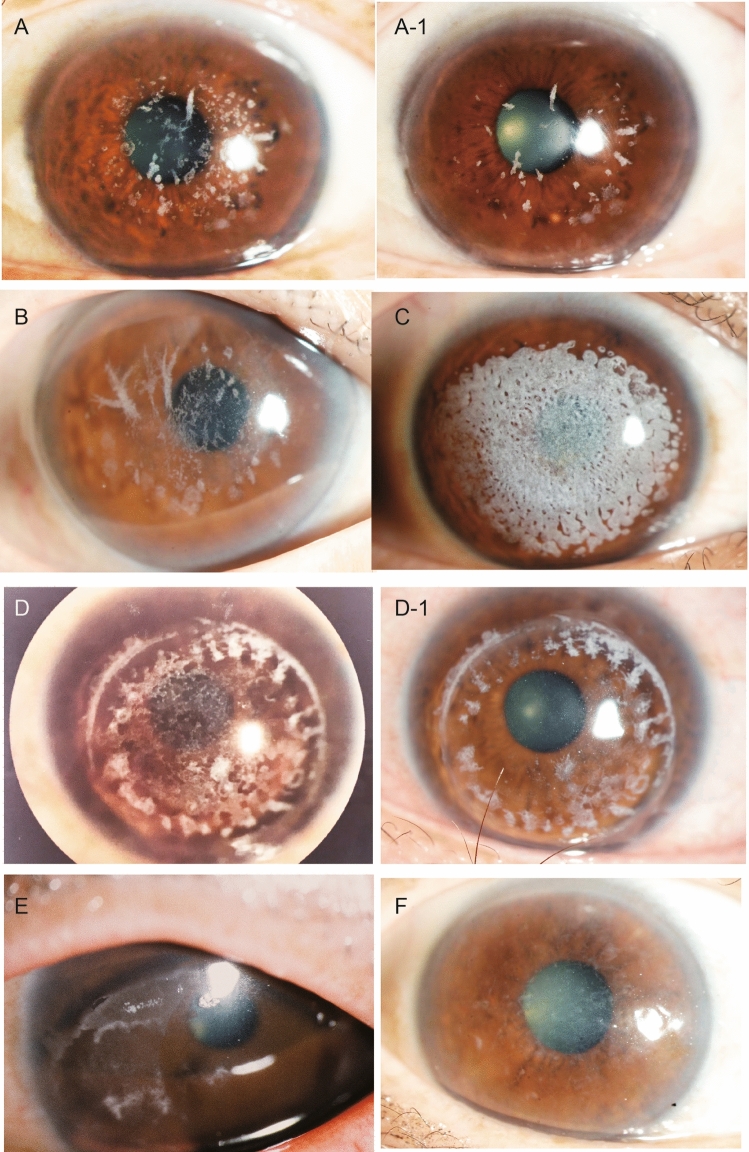
Slit-lamp microscopy photographs of the anterior segment at 18.5 years initial phototherapeutic keratectomy (PTK) for hetero-type granular corneal dystrophy (GCD) prior to re-excimer laser PTK (re-PTK) (**A**) and post 5 years (**A-1**). The photographs at 20 years initial PTK for hetero-type GCD prior to re-PTK (**B**).These 2 eyes had the longest elapsed time period between the two PTK operations. The photographs of 46 months (**C**) and 31 months (**D**) after the initial PTK for homozygous-type GCD before re-PTK. The photographs at 6 months initial PTK homozygous-type GCD post re-PTK (**D-1**). Images showing the pre-re-PTK findings of band keratopathy (**E**) and lattice corneal dystrophy (**F**).

**Figure 3:**
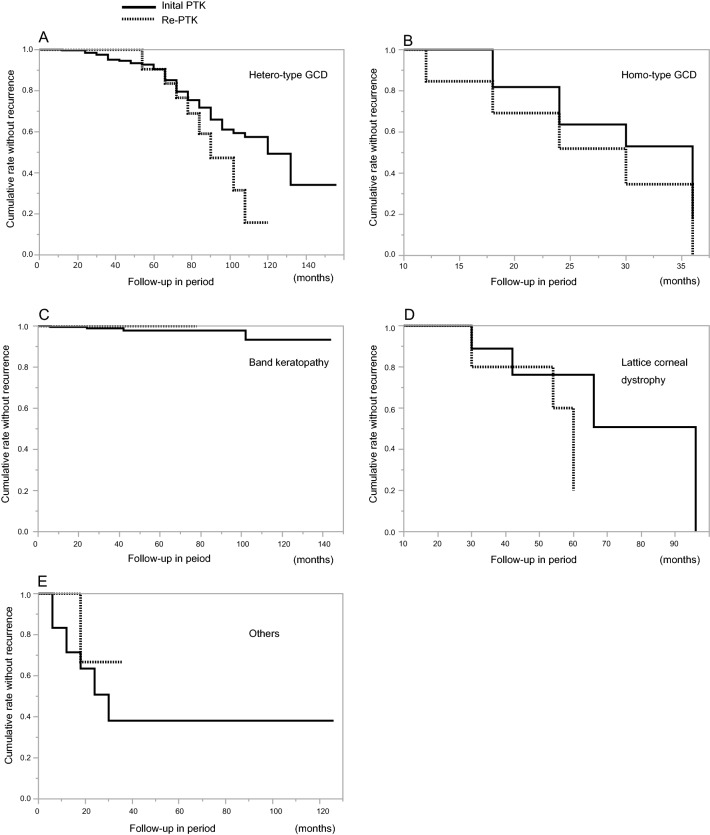
Graph showing the survival curve of the non-recurrence of disease after re-PTK compared with initial PTK among the 5 groups. There was no difference in the recurrence rate after re-PTK and initial PTK among the 5 groups.

The eyes with recurrent hetero-GCD (Fig. 2A,B) were treated by re-PTK, and the mean change of CDVA in those eyes was − 0.27 ± 0.33 logMAR (range, -1.70 to 0.24 logMAR). CDVA improved significantly after surgery (*P* < 0.0001). After recurrence, GCD gradually progressed for several years, which eventually led to a decrease in VA. No significant difference was found between the recurrence rate post-re-PTK and that post initial-PTK (Kaplan–Meier-method analysis, *P* = 0.20) (Fig. 3A).

The eyes with recurrent homo-GCD were treated by re-PTK (Fig. 2C,D), and the mean change of CDVA in those eyes was − 0.85 ± 0.39 logMAR (range, − 1.35 to − 0.10 logMAR) (*P* < 0.0001). No significant difference was found between the recurrence rate of homo-GCD post-re-PTK and that post initial PTK (Kaplan–Meier-method analysis, *P* = 0.24) (Fig. 3B). The recurrence occurred at approximately 6-months after re-PTK, followed by an increase of opacity. Five eyes of 4 patients had undergone PK prior to the initial PTK, however, there was no significant effect on the recurrence rate of PTK, or not, after PK.

The number of recurrent BK was 7 eyes in 7 patients (Fig. 2E), and the mean change of CDVA in those eyes was − 0.24 ± 0.16 logMAR (range, -0.48 to 0 logMAR) (*P* = 0.008). No significant difference was found between the recurrence rate of BK post-re-PTK and that post initial-PTK (*P* = 0.80) (Fig. 3C). Moreover, there were no cases of disease recurrence after re-PTK for BK.

In the LCD group eyes (Fig. 2F), the mean change of CDVA was -0.61 ± 0.28 logMAR (range, − 0.92 to − 0.18 logMAR) (*P* = 0.003). No significant difference was found between the recurrence rate of LCD post-re-PTK and that post initial-PTK (*P* = 0.16) (Fig. 3D). Moreover, no significant difference was found between the recurrence speed in the other-disease group post-re-PTK and that post initial-PTK (*P* = 0.46) (Fig. 3E), and CDVA was also improved in the other-disease group post-re-PTK (*P* = 0.02).

In all subjects, no sight-threatening adverse events occurred. Mild circular haze was observed in some of the cases, however, there were no cases that required another PTK for that opacity. Treatment with mitomycin C (MMC) was used only in 3 homo-GCD eyes that underwent re-PTK. Hyperopic shift was observed, and the mean change in spherical equivalent refractive errors between pre and post-re-PTK in 24 eyes was + 1.77 diopters (D) (range, − 2.25 to + 5D). The hyperopic shift was correlated with ablation depth (P = 0.0006), more ablation depth caused more hyperopic shift. VA decreased by two lines or more in only 1 hetero-GCD eye after re-PTK, and that patient had a cataract and was referred to another hospital for cataract surgery at 1-month post-re-PTK.

## Discussion

All disease groups showed significant improvement in CDVA after re-PTK. However, compared with the hetero-GCD cases, there was a significantly greater rate of disease recurrence in the homo-GCD cases after re-PTK. There was no difference in recurrence rate between post-re-PTK and post-initial PTK.

To date, there have been a few published case reports on the rate of disease recurrence following re-PTK^16,17^. In theory, repeated laser irradiation activates the stromal cells into fibroblasts, which increases the production of collagen and corneal opacity. As the number of PTKs being performed has increased, this is reportedly a trend towards a shorter elapsed time to re-operation^18^. In our experience, the elapsed time to recurrence in all disease groups is equal for re-PTK and initial PTK. It should be noted that most of the re-PTK surgeries at our institutes were performed 3 or more years after the initial PTK. The cornea stabilized after a period of time, re-PTK might make equal response with initial PTK. Recurrent corneal dystrophy and degeneration can be treated with corneal transplantation. However, due to the large postoperative astigmatism and the complications of infection and rejection, corneal transplantation should be an option when visual improvement cannot be achieved with re-PTK.

Performing a re-PTK does not have a negative effect on the cornea. In fact, the excimer laser has been used clinically for more than 20 years as a laser that can ablate corneas without opacity with proper prophylaxis^19^. It has been reported that the cornea after excimer laser ablation maintains a stable morphology^20^ and corneal shape^21^ for a long period of time. It is also known that the corneal surface normalizes after PTK^22^ and that, except for some CDs, disease recurrence occurs slowly^4,6^. Based on our observations and the fact that the ablation depths of the first and second resections were almost the same, the location of the recurrent corneal opacities are same as the depth of the original diseases. The mean ablation depth in the first PTK, granular corneal dystrophy was 106.7 μm and band keratopathy was 100.3 μm. In this re-PTK, granular corneal dystrophy was 111.5 μm and band keratopathy was 96.7 μm, with no obvious difference. After a sufficient period, the second PTK should not affect the cornea any differently than the initial PTK.

Compared with the postoperative outcomes post initial PTK, the same side effects and complications can occur post-re-PTK. PTK causes a large epithelial defect, so caution is needed in regard to corneal infection. Our findings showed that hyperopic shift after PTK occurred at the same level as after the initial PTK. Due to a rapid recurrence of homo-GCD in 3 eyes, MMC was applied during surgery to gain the effect to slow down the recurrence^23,24^, not to prevent corneal haze^25^. In all cases that underwent re-PTK, there was no haze affecting the VA post surgery. Moreover, the use of MMC was generally not necessary for re-PTK.

In this study, two-thirds of the hetero-GCD cases, one-half of the homo-GCD cases, and all of the LCD and other disease cases were not genetically tested. Other than in the homozygous and heterozygous types of GCD, the effect of genotype on relapse was unknown. In some cases of inflammation, such as conjunctivitis, the opacity recurred quickly, and the risk factors for recurrence for each disease will be examined in greater detail in the future.

In conclusion, re-PTK was found to be safe and effective for treating disease recurrence following an initial PTK. Statistically, we found that the elapsed time to disease recurrence following re-PTK was nearly the same as that post initial-PTK. Thus, re-PTK should be considered after the recurrence of the disease.
